# The other side of the story – maternal perceptions of safety advice and information: a qualitative approach

**DOI:** 10.1111/cch.12224

**Published:** 2015-01-20

**Authors:** J. Ablewhite, D. Kendrick, M. Watson, I. Shaw

**Affiliations:** ^1^ School of MedicineUniversity of NottinghamNottinghamUK; ^2^ Faculty of Medicine and Health SciencesQueens Medical CentreUniversity of NottinghamNottinghamUK; ^3^ School of Sociology and Social PolicyUniversity of NottinghamNottinghamUK

**Keywords:** advice, children, injury, qualitative, safety

## Abstract

**Background:**

A qualitative study of maternal perceptions of home safety advice. The aim was to gain an understanding of maternal perceptions of and possible barriers to the implementation of home safety advice.

**Methods:**

Semi‐structured interviews with 37 mothers with a child aged less than 5 years of age; 16 were mothers living in an area of socio‐economic disadvantage (with a high rate of childhood unintentional injury), 21 were mothers living in an area of relative affluence (with a low rate of childhood unintentional injury). Thematic analysis was used to analyse the data.

**Results:**

Although some mothers living in both areas found talking to a health professional about child home safety was helpful, mothers in both areas tended to find talking to other mothers as being more helpful and they preferred this to talking to a professional. Barriers to obtaining safety advice from professionals exist for mothers living in both areas. Mothers living in the advantaged area describe ‘feeling silly’ and that they should ‘know it already’ when talking to professionals. Mothers living in the disadvantaged area are less likely to access home safety advice due to fear of being perceived as an incompetent mother and the fear of social service involvement.

**Conclusions:**

Mothers find home safety advice from other parents more useful and prefer this to advice from professionals. This suggests greater use could be made of appropriately trained parents to deliver safety advice and education. Fear and mistrust can limit access to child safety advice in parents living in disadvantaged areas and this may be a potential explanation for differential unintentional injury rates as those who need the advice and support most may be least likely to access it. Further research should explore how professionals can build trust, gain parents' confidence and provide child safety advice and education that is targeted appropriately to parents living circumstances and their child safety needs.

## Introduction

Unintentional injuries are a major public health issue affecting children in England today. In England, between 2008 and 2012, unintentional injuries resulted in 311 deaths in children under 5 years, making this the most common cause of death (Public Health England 2014). More than 45 000 children under 5 years were admitted to hospital in England in 2012/2013 (Health and Social Care Information Centre 2013), in addition, unintentional injury in children under 5 years accounted for approximately 450 000 emergancy department attendences in the UK (Department for Trade and Industry 2003). For children aged 0–4 years of age, unintentional injuries are most likely to occur within the home environment, as that is where they commonly spend the majority of their time (Department of Health 2002).

Within the UK, unintentional injuries in children are not evenly distributed across society. There are wide inequalities between different social groups in relation to those experiencing injury mortality and morbidity (Towner 2002). Children living in socio‐economic disadvantage are more likely to suffer unintentional injury than children from more advantaged backgrounds (MacKay *et al*. 1999; Hippisley‐Cox *et al*. 2002; Laflamme *et al*. 2010).

The delivery of safety advice to parents of young children is one approach that can be used to help to reduce unintentional injury rates. The provision of safety advice is a key element of England's national child health surveillance programme (Department of Health 2008) and the National Institute of Health and Care Excellence recommends health and social care staff provide child‐focused home safety advice and if appropriate, referral for home safety assessments and safety equipment provision (NICE 2010).

A systematic review of health professionals' knowledge, attitudes and current practices in relation to childhood unintentional injury identified key points relevant to the delivery of safety advice to parents (Woods 2006). Firstly, it should not be assumed that health professionals, working with children and families, will be knowledgeable about child unintentional injury prevention. Secondly, health professionals recognize the importance of and are keen to be involved in injury prevention work. However, health professionals may not give unintentional injury priority in comparison with other health priority areas. Barriers to effective child unintentional injury practice for health professionals, included lack of training and knowledge, time constraints because of the pressure of caseloads and covering vacant posts.

A systematic review of qualitative studies identified barriers to the delivery of safety advice (Smithson *et al*. 2011). A major barrier to child injury prevention work, with young mothers on low incomes, related to a mistrust of professionals, with mothers feeling anxious about the consequences of talking to professionals. For example, talking to a health professional may lead to their child being seen as at risk, or removal of the child from them following an accusation of abuse or neglect. A systematic review of barriers and facilitators for child injury prevention interventions identified that a facilitator for the delivery of safety advice was using child health professionals, or other professionals, such as parent educators, to deliver safety messages as they were a trusted familiar figure with established relationships with families (Ingram *et al*. 2012).

An important difference between the perspectives of professionals and parents in relation to safety advice was highlighted in the findings of (Roberts *et al*. 1995). Professionals tended to describe an individualistic approach to injury prevention, for example, that parents lacked knowledge about how to prevent child injuries, which resulted in children being put at risk of injury. The parental perspective was quite different from that of the professionals. Parents described feeling patronized, or that health professionals did not understand the reality of their daily lives so the safety advice provided was not practical. Parents were able to suggest, and had implemented, imaginative and low‐cost ideas that met their child safety needs (Roberts *et al*. 1995).

In addition to safety advice from health professionals, parents may turn to other sources for safety advice or information. The Staying Safe Survey (Department for Children Schools and Families 2010) identified the three most common sources of safety advice and information cited by parents, to be health visitors/midwives (40%), family members (39%) and friends/other parents (29%). The survey also found that 35% of parents in lower socio‐economic groups (with an income less than £20 000) wanted more safety advice but only 34% had asked for it.

A recent qualitative study explored mothers' views about the best method for designing interventions to deliver home safety advice (Khanom *et al*. 2013). Their findings suggest delivering such advice through a range of sources including social networks, suitably trained mothers and health professionals, from pregnancy and then in line with the ages and stages of child development.

Existing evidence regarding the delivery of safety advice has tended towards exploring professional perspectives (Woods 2006; Watson *et al*. 2007). The aim of this study was to gain an understanding of maternal perceptions of, and possible barriers to the implementation of safety information and advice and how these varied between families living in a disadvantaged and a more affluent area.

The study was carried out in two wards in Nottingham, UK. St Ann's ward is one of the most deprived wards within Nottingham, with a high level of transience and an area where significant social problems exist. Social problems within the ward include a high number of people living on low incomes, poor quality housing, reduced life expectancy and higher injury rates than other wards in Nottingham and much higher than the national average (Nottingham Primary Care Trust Annual Health Report 2003–2004). By comparison, Wollaton West ward has a low level of transience and the population comprises people in professional employment, good quality privately owned homes and a low rate of child injury.

## Methods

Recruitment was undertaken in three stages. Firstly, health visitors, working in each area, were invited to assist with the recruitment of parents to the study. Secondly, health visitors that agreed to take part were asked to send a participant recruitment pack to 50 parents. To ensure that cases were selected systematically, they were asked to select every other child in a given age bracket. The age brackets were 0–11 months, 12–23 months and 24–48 months. Parents who were interested in taking part were asked to return a reply slip with their contact details in a prepaid envelope to the researcher. Thirdly, the researcher contacted parents that had responded, by telephone and if they agreed to take part in the study a date and time was arranged for the researcher to visit them at their home. The aim was to recruit 21 parents in each ward, seven with a child in each age bracket. The use of quota sampling ensured an even distribution of child ages between the wards (Patton 2001). Because of a low response rate from mothers using the postal method, a different recruitment avenue was sought. In order to complete the St Ann's sampling frame, additional recruitment took place via a mother and toddler group and via a children's centre.

Data collection included in‐home interviews that lasted on average approximately 40 minutes. One interview took place at a children's centre, at the request of the participant. All interviews were conducted with the mother.

A semi‐structured interview schedule was developed, based on findings of previous studies (Santer & Stocking 1991; Department for Children Schools and Families 2010; Smithson *et al*. 2011). The interview schedule was piloted with two families; minor adaptations were made to the guide following the pilot. Interviews were audiotaped, with written consent and transcribed. During transcription, data were anonymized, and the interviews were transcribed verbatim.

The data analysis process was developed as follows: the data were explored for emerging themes, three researchers read the transcripts noting significant themes (Silverman 2000). Following this, the researchers discussed the main emerging themes and sub‐themes and developed coding categories. Definitions for the coding categories were then agreed. The coding process of the interviews included identifying both confirming and disconfirming cases (Murphy *et al*. 1998). Themes and sub‐themes were used to code the data using the computer software package Nvivo (QSR International). As the data were analysed, any emerging themes were applied to previously coded data.

## Results

A total of 37 mothers took part in the study. In Wollaton West, 33 (22%) responses were received from the first mailing of 150 recruitment packs and 21 interviews were conducted. In St Ann's, 13 (8%) responses were received from a first and reminder mailing to 150 mothers and 10 agreed to be interviewed. Because of a low response rate from mothers using the postal method, a different recruitment avenue was sought. Recruitment of mothers through a St Ann's children centre and a local mother and toddler group resulted in seven parents agreeing to be interviewed, one of whom withdrew prior to interview. When 16 mothers had been interviewed in St Ann's, the decision was taken to cease recruitment because of data saturation. The characteristics of participants in the two areas are shown in Table 1. The table shows there were important differences between St Ann's and Wollaton West in terms of maternal age, marital status, housing tenure and employment. There were no differences between the mothers in St Ann's recruited via the different recruitment strategies.

**Table 1:** Participant characteristics

	St Ann's ward	Wollaton West ward
Maternal age		
Teenagers	2 (12.5%)	–
20–29	12 (75%)	–
30–30	2 (12.5%)	17 (81%)
40–49	–	4 (19%)
Marital status		
Lone parent	10 (63%)	1 (4%)
Two‐parent household	6 (37%)	20 (96%)
Housing tenure		
Council rented	13 (82%)	–
Private rented	1 (6%)	–
Private owned	2 (12%)	21 (100%)
Employment		
Household does not have a parent in paid employment.	16 (100%)	–
Household has at least one parent in paid employment.	–	21 (100%)
Household has two parents in paid employment.	–	16 (76%)
Injuries disclosed during interview		
Minor trips/falls	1 girl	15 (8 boys 7 girls)
Fall down stairs	4 (2 girls, 2 boys)	–
Fall from furniture	2 boys	–
Scald	2 (1 boy, 1 girl)	–
Minor burn	1 boy	2 (1 boy, 1 girl)
Poisoning	1 boy	–
Poisoning because of medication	1 girl	–

The results of the analysis are presented below according to themes that emerged from the data: safety advice and safety equipment, fear lack of trust and feeling silly, impractical safety advice, feeling patronized, learning from other family members, safety advice that was useful. Quotes are labelled as ‘SA’ being a mother from St Ann's and ‘W’ being a mother from Wollaton West.

### Talking to professional about child safety

#### Safety advice and safety equipment

A key difference between mothers living in Wollaton West and St Ann's was that mothers living in Wollaton West describe having a greater level of control over adapting their home to meet the safety needs of their child than mothers living in St Ann's. This was largely due to the fact that the majority of mothers living in St Ann's were living in rented or social housing and had limited financial resources to purchase safety equipment. At the time of the study, St Ann's was an area that had a home safety equipment scheme, which provided free safety equipment and home safety advice for families on low incomes. Eight of the parents in the study from St Ann's had received equipment and advice from the safety equipment scheme, and these parents described the equipment as useful. In addition, recipients of safety equipment seemed more receptive to safety advice from the people who installed it for them, than from health professionals.

When [daughters name] was small Sure Start did a promotion that if you signed up with Sure Start they gave us three stair gates, smoke alarm, plug sockets, cupboard locks … There was a bloke that came round and he fitted the stair gates properly so we got the one at the bottom of the stairs one at the top and one through to the kitchen. And they explained how to fit the cupboard locks and they fitted the cupboard locks under the kitchen sink and said if it drops and showed us what to do and said if we needed or any advice just to give them a ring. (SA15 boy 0–11 months girl 24–48 months)

You definitely have to have a stair gate at the top of my stairs. … Sure Start put a stair gate at the top … and my fire alarms, if they didn't give me them I don't think I'd have a fire alarm and stair gates if they didn't offer me that advice and give me them for free I don't think I would ever have them and they're life savers they are. (SA4 boy 24–48 months)

In contrast, Wollaton West, being a relatively affluent area, did not have a home safety equipment scheme. Ten parents (48%) living in Wollaton West describe finding out about safety equipment to reduce child injury risks by browsing around shops such as Mothercare and purchasing the safety equipment that they perceive will meet the changing safety needs of their child.

It's a learning curve but also every parent and baby shop like Mothercare for example you go to I mean they have a whole section on safety so even by looking at that it will tell you what you need. (W16 boy 24–48 months)

#### Fear, lack of trust and feeling silly

An important difference between mothers living in the two areas was found with regard to talking to professionals about child safety. Nine (56%) of the parents living in St Ann's described a fear of talking to a professional because of a lack of trust of professionals and the potential consequences of asking for advice. In contrast, five (23%) mothers living in Wollaton West described feeling silly or that they should ‘know it’ already.

I mean they can take you the actual wrong way and they'll get the social services involved. So I don't really talk to them. I'd rather ask Sure Start you know [leaders name] and them lot. … Like with a health visitor you put him on the bed for a minute and tell the health visitor that he fell off they'll like shout at ya and say why did you put him on the bed and they don't know what the situation was like you quickly put them on the bed. And it's just I don't trust them. (SA14 boy 12–23 months)

They (Health visitors) think it should come to them (parents) naturally and they shouldn't have to ask. When she was a baby and I had to give her a bath I didn't know which position to make her sit but I didn't ask anybody because I was too ashamed that everybody should know how to give a baby a bath. (W14 girl 24–48 months)

#### Impractical safety advice

Three mothers living in St Ann's and three living in Wollaton West described how safety advice given by their health visitor was difficult or impractical to incorporate into their everyday lives. In St Ann's one example includes a mother being advised that she needed safety equipment for her home but she did not have the financial resources to implement the advice.

They (the health visitors) say you need this equipment and that equipment, and I mean if you haven't got the money then how can you enforce what they're telling ya and stuff. I mean they do give you some help but they gave me one gate and it's like where shall I put it. They say you need a gate at the top of the stairs, they say you need a gate at the bottom of the stairs, you need one on the kitchen so it's kind of hard. For me to buy stair gates and a fire guard it's a lot of money. I could have done with a fire guard but she don't touch it. But some people need help straight away cos it could be life or death. (SA12 girl 24–48 months)

I mean I've had different advice with my second than with my first and that's only two years. Things about feeding them and it just changes so much that you just kind of think I'm just gonna get on with it. … Some of the advice they give you is just irrelevant and not practical and that's when you kind of think well I'm not going to listen to anything you say now because you've said that it just makes me think well you don't know what you're talking about, you don't know what it's like on a day to day basis so is anything you tell me going to be relevant and you do you just switch off. (W9 girl 0–11 months, boy 24–48 months)

#### Feeling patronized

One mother living in St Ann's and one mother living in Wollaton West described feeling patronized by the health visitor. The St Ann's mother described feeling patronized when the advice they were given was ‘textbook’ and not ‘real life’. In Wollaton West, the mother described feeling patronized and receiving advice that was ‘useless’ with a first child and so was resistant to safety advice with her second child.

Well we had a baby walker right … and the health visitor said you just want to throw that away. I mean she said it like we were stupid but of course we weren't going to have it up stairs or near the ironing board I mean its common sense … and the health visitor and the midwife both talked like its just straight out of a text book but its not like that and you find your own way and if you're not doing it how they say with their text book then you're wrong … and she made you feel like about that big. (SA3 boy 12–23 months, boy 24–48 months)

To be honest I think we felt quite patronised by it … I just think that I had to figure it all out myself with my first child so I don't, I really don't need it second time round. So don't even try giving it to me second time round because nobody was there to give it to me for my first one. And I've got a three year old whose quite healthy thank you very much, I've sussed it out myself … Well the only time I actually went to a health visitor with my first child I was given such useless information that I guess in my own mind I just thought I'll stick two fingers up at the establishment. (W12 boy 24–48 months, girl 0–11 months)

#### Safety advice that was useful

During the interview, mothers were asked if they could recall any home safety advice that they had found useful. One mother in St Ann's and one in Wollaton West recalled safety advice from a health professional that was useful. In the response of the St Ann's mother, she described how being given leaflets was helpful as it can be difficult to absorb information received during the contact with the health visitor. This account also describes how information presented in leaflets needs to be easy to read and sensitive to those with poor literacy skills.

The health visitor would tell me things and give me leaflets. Cos sometimes you can go in there and you are feeling quite frustrated anyway. There's lots of people in there you've had to wait a long time and then finally it's your turn. You've forgotten half what you've gone in for anyway, your daughters screaming cos she doesn't want to be there and so you're kind of stressed. You can't really absorb the information that well. The health visitor down there's lovely and she gave me lots of leaflets so when I did come away although I did think ‘what's she said?’, at least I had the leaflets to refer back to and I think that's important. Especially at the beginning and they're only a few months old and you hardly get any sleep. I think they do need to give you things that you can take away rather than just information you absorb it and good luck with it kind of thing … you need to make sure information is there but information that is easy to read. I mean a lot of people I mean I've got friend that they're not very good with their literacy skills that it's embarrassing when you go to the health visitor because the last thing, that's on your mind, you don't want them to think you're not doing a very good job. (SA6 girl 24–48 months)

### Talking to other parents about child safety

One of the themes that emerged was that rather than talking to professionals about child safety, mothers living in both areas, nine (56%) in St Ann's and 10 (48%) in Wollaton West described talking to friends or family as an important source of safety information and advice. Mothers living in St Ann's also appeared to have smaller social networks, more often describing talking to individuals than mothers living in Wollaton West, who more commonly described talking to groups of friends or other parents.

I think you talk about things you might feel a bit dodgy about that you wouldn't raise to a professional you would raise that to another Mum but not to a professional. I'm more on the side of I'll figure it out myself or I'll ask my mum or my brother cos he's got a couple of kids. (SA10 boy 24–48 months)

I've got a few friends and you go round to their houses and they've all got their guards on and bits and bobs so yeah you do learn from that. And when you discuss accidents you think I'll keep an eye out for that. (W11 boy 0–11 months, girl 24–48 months)

## Discussion

This paper presents the key findings of a qualitative study exploring maternal perceptions of home safety advice and potential barriers to implementing such advice in their day‐to‐day work to keep children safe within the home.

Although some mothers living in both areas found talking to a health professional about child home safety to be helpful, parents in both areas tended to find talking to other parents more helpful and they preferred this to talking to a professional. Some parents found advice from professionals impractical or difficult to incorporate into their circumstances. Barriers to obtaining safety advice from professionals exist for parents living in both areas. Mothers living in the advantaged area described ‘feeling silly’ and that they should ‘know it already’ when talking to professionals. Mothers living in the disadvantaged area described mistrust of professionals and fears of social service intervention if they are perceived to be an incompetent mother.

There are several strengths to this study. Our findings add to the sparse literature on maternal attitudes, perceptions and experiences of home safety advice and highlight the importance of ensuring safety advice is appropriate to the needs of individual families. The findings are important as there is little existing research, conducted within the UK, relating to mothers' perspectives of talking to professionals about child safety. Providing health visitors with specific criteria for selecting mothers to invite to the study minimized the potential for selection bias. Additional recruitment strategies used in St Ann's ensured that sufficient participants were recruited to achieve data saturation, despite this being a ‘hard to reach’ group. Finally, a rigorous approach to data analysis was used, with three researchers identifying and discussing themes in the data to avoid subjectivity (Pope *et al*. 2000). Data that did not fit into the main themes was also included (Murphy *et al*. 1998).

The limitations of this study are that it is possible that the parents who agreed to take part in the study had a particular interest in or were motivated by the aims of the study or child safety in general and the data may, therefore, not be representative of parents living in the two areas (Bowling 2002). It is not appropriate to make generalizations to the wider population from the findings of this study. However, it may be possible to transfer the findings to similar groups living in similar circumstances.

There are some limited examples of parents living in both areas who found talking to a health visitor helpful with regard to child safety. However, an important difference between the two areas related to mistrust of professionals and fear of the consequences of talking with them about child safety. The finding is consistent with other studies that young mothers on low incomes living in disadvantaged areas described an anxiety and mistrust of talking to professionals (Smithson *et al*. 2011). Such mistrust and fear may exacerbate differentials in injury risk, as families who may have the greatest potential to benefit from safety advice and support from professionals may be least likely to access it.

In addition to safety advice from health professionals, parents turn to other sources for safety advice or information, including family members, friends or other parents (Department for Children Schools and Families 2010; Khanom *et al*. 2013). Our study found that parents living in both areas discuss child safety with family members and friends and that this was an important source of support for many. For some parents, this information was more helpful than that from health professionals. In addition, parents found that information from other parents was more practical and easier to implement in the context of their everyday lives than that provided by professionals. As parents in the disadvantaged area appeared to have smaller social networks for obtaining such information than those in the advantaged area, this may again mean, those families who may benefit most from safety advice have the least access to it. Mothers' preference for obtaining safety advice from parents, family members or friends is important for injury prevention practitioners. It demonstrates the potential for increasing trust in the relationship between mothers and professionals and the need for professionals to provide context specific and appropriate safety advice, tailored to the needs of each family. There is no ‘one size fits all’ and training for professionals needs to address the complexity of providing safety advice, especially in the context of maternal fear and mistrust.

Although advice from family and friends may be highly useful and relevant, at times it may also be out of date, misinformed or not take account of legislative developments or shifting societal expectations about safety behaviour. In addition, advice from family members and friends may be based on individual experiences relating to their own child rather than a broader understanding of the ages and stages of child development and potential injury risks associated with the different stages (Agran *et al*. 2003; Pickett *et al*. 2003). Peer programmes, where parents are trained to provide home safety advice to other parents, for example, lay home visiting programmes, have demonstrated reductions in injury risk (Barlow *et al*. 2006). Delivering home safety advice through social networks and appropriately trained mothers has been suggested (Khanom *et al*. 2013). Our findings support the use of these approaches.

Further research is required to explore mothers' experiences of talking with professionals about child well‐being and child safety. Further research should explore how professionals can build trust, gain parents' confidence and provide child safety advice that is targeted appropriately to parents living circumstances and their child safety needs.

## Conclusion

It is necessary to address the barriers perceived by mothers with regard to talking to health professionals about safety. Advice given to families must take account of the environmental and economic circumstances in which they live so that it is realistic and practical.

As mothers find safety advice from other parents more useful, and prefer this to advice from professionals, this suggests greater use could be made of suitably trained parents and social networks to deliver safety advice in line with the ages and stages of child development.

Key messages

- Barriers exist for parents with regard to obtaining safety advice from professionals.
- Greater use could be made of suitably trained parents and social networks to deliver safety advice.
- Safety information needs to be tailored to the needs and circumstances of individual families.

## Ethics

Ethical approval was provided by Nottingham research ethics committee Reference Number 06/Q2403/ 65. Organizational approval was granted by Nottingham Primary Care Trust.
